# A High-Precision Time-Varying Survival Model for Early Prediction of Patient Deterioration: A Retrospective Cohort Study

**DOI:** 10.3390/jcm15051690

**Published:** 2026-02-24

**Authors:** Nishchay Joshi, Brian Wood, David Chapman, Martin Farrier, Thomas Ingram

**Affiliations:** Wrightington Wigan & Leigh Teaching Hospitals NHS Foundation Trust, Wigan WN1 2NN, UK; nishchay.joshi@wwl.nhs.uk (N.J.); brian.wood@wwl.nhs.uk (B.W.); david.chapman@wwl.nhs.uk (D.C.); martin.farrier@wwl.nhs.uk (M.F.)

**Keywords:** clinical deterioration, patient deterioration, early-warning system, high-risk patient, survival analysis, Cox proportional hazards model, machine learning, NEWS2, alert fatigue, electronic health records

## Abstract

**Background:** Clinicians rely on clinical judgement and vital sign monitoring to identify patient deterioration, commonly supported by systems such as the National Early Warning Score 2 (NEWS2). However, NEWS2 is associated with a high false-positive burden, contributing to alert fatigue in increasingly pressured clinical environments. Consequently, there is a growing need for early warning systems (EWS) that not only detect deterioration but do so with higher precision to prioritise clinically meaningful alerts. We aimed to develop and validate a prognostic EWS capable of predicting real-time clinical deterioration in hospitalised adult patients. **Methods:** We conducted a retrospective observational cohort study using routinely collected Electronic Patient Record (EPR) data. A Cox proportional hazards model with time-varying covariates was developed to estimate dynamic risk of deterioration. Deterioration was defined as unplanned transfer to intensive care, unplanned surgery, or in-hospital death. Data for model development comprised 37,989 adult inpatient episodes admitted between January 2022 and October 2024, and were initially split into training, temporal validation and test datasets. An extended evaluation period included 11,048 patients admitted through September 2025. Model performance was compared with NEWS2 at the emergency-response threshold (≥7). **Results:** The final model produced a tiered “traffic-light” risk profile and demonstrated substantially higher precision than NEWS2 while maintaining comparable recall in our test data. At the red alert threshold, precision was 60% compared with 16% for NEWS2 ≥7, with 82% versus 43% of alerts occurring within 24 h of deterioration. Performance remained consistent across the extended evaluation period. **Conclusions:** A survival-based EWS incorporating time-varying covariates achieved higher precision and improved temporal alignment with deterioration events compared with NEWS2. A tiered amber–red alert framework may support more targeted escalation, reduce alert fatigue, and enhance early identification of clinical deterioration.

## 1. Introduction

Multiple studies have shown that early detection of deterioration and early intervention in ward patients can significantly improve patient outcomes and potentially save lives [1,2]. Early identification of deterioration followed by timely intervention can also shorten a patient’s length of stay in the hospital and avoid intensive care unit (ICU) transfers leading to reduced resource utilisation and substantial cost saving for the hospital [3,4].

Deteriorating physiological parameters are often present in patients around 24 h prior to ICU transfer/in-patient death, which may sometimes go unnoticed [5]. To avoid this, various early warning systems (EWS) are used worldwide to track patient vitals and detect clinical deterioration. The National Early Warning System 2 (NEWS2) was developed based on expert opinion, incorporating clinical experience, to identify key physiological parameters indicative of patient deterioration. It is the standard system recommended by the Royal College of Physicians and is used for monitoring deterioration in adult patients across NHS England [6]. NEWS2 score is calculated based on the vital signs of the patient and does not include other potentially important variables, such as the patient’s age, frailty, and laboratory test results.

Despite its widespread use, both published evidence and clinician experience indicate that NEWS2 generates a high number of false positives. Rather than signalling only acute deterioration, the system also repeatedly alerts for persistently abnormal but clinically stable observations. For example, a patient admitted with a chronically high respiratory rate may continuously trigger a high NEWS2 score despite showing no new clinical decline. Such repeated alerts place a substantial cognitive and emotional burden on clinical staff. Frequent false alarms increase workload, interrupt clinical tasks, and erode trust in early warning systems, leading to clinicians discounting or delaying responses to alerts. This phenomenon of alert fatigue has been widely recognised across clinical decision support systems [7]. Unnecessary escalation due to inappropriate alerting may also negatively affect patients. Recurrent monitoring, overnight observations, and repeated clinical reviews can disrupt sleep, increase anxiety, and contribute to a perception of clinical instability even in the absence of true deterioration.

There is potential to improve early detection of deterioration by using real time patient-specific data collected by the Electronic Patient Record (EPR) system. Indeed, alternative EWS have recently been developed, such as the Rothman Index [8], which takes a data-driven approach using statistical modelling to create a composite score, and eCARTv5 [9], which uses discrete time survival analysis to predict deterioration, and have been incorporated alongside NEWS2 in healthcare settings. Machine learning techniques have also gained popularity in attempting to create a system that tracks patient data in real time and raises an alert when deterioration is detected. For example, the Advanced Alert Monitor score was created using a discrete-time logistic regression model [10].

The objective of this study was to build upon this literature of bespoke EWS by developing a novel prognostic tool to detect patient deterioration in real time, issue alerts with higher precision than NEWS2, and thereby reduce alert fatigue burden for clinicians. This report outlines the data pre-processing steps, model development, and comparison of its performance to NEWS2. Additionally, the report will address the limitations, potential biases, and future enhancements to improve the model’s predictive capabilities.

## 2. Materials and Methods

### 2.1. Study Setting

This retrospective observational cohort study was conducted at Wrightington, Wigan and Leigh (WWL) Teaching Hospitals NHS Foundation Trust, UK, using data from adult inpatients admitted to the general medical and surgical wards of the Royal Albert Edward Infirmary, Wigan. The study received institutional approval in accordance with WWL research governance and ethical regulations (study reference: Corp/SE/2025-26/14). This study was reported in accordance with the STROBE guidelines for observational cohort studies and the TRIPOD guidelines for prognostic model development and validation.

### 2.2. NEWS2

National early warning system 2 (NEWS2) is the system recommended across NHS England and is currently used to assess the severity of a patient’s condition and detect potential clinical deterioration [6]. It assigns scores based on six physiological parameters: respiratory rate, oxygen saturation, systolic blood pressure, heart rate, level of consciousness, and temperature. Each parameter is scored between 0 and 3 based on deviation from normal values. A total NEWS2 score is calculated by summing the scores for each parameter; the higher the total NEWS2 score, the greater the clinical risk.

The frequency of monitoring patient vital signs increases as the NEWS2 score rises. A NEWS2 score of 7 or higher warrants an emergency response and continuous monitoring of vital signs. In this study, NEWS2 thresholds of 7 and above are used to evaluate performance against the proposed model.

### 2.3. Data Collection

Data were sourced from the EPR (Sunrise, Altera Digital Health, Raleigh, NC, USA) at WWL, a comprehensive electronic health record platform that stores detailed patient information. The EPR contains patient demographic information (e.g., sex, age), clinical information (e.g., specialty, frailty), vital signs, laboratory results, and time-stamped records of key events such as admissions, discharges, ward transfers, surgery, etc. Patient observations and events are stored in the EPR in real time as they are recorded in the ward.

The study population comprised all adult patients (age ≥ 18 years) admitted to acute medical and surgical wards between 1 January 2022 and 31 October 2024. Only patients with a completed hospital stay were included in the analysis. Maternity patients and patients on end-of-life care were excluded.

We included three categories of outcome marker for patient deterioration: (i) transfer to the Intensive Care Unit (ICU) or High Dependency Unit (HDU), excluding patients admitted directly from Accident and Emergency (A&E); (ii) unplanned surgery, defined as emergency surgical intervention due to clinical deterioration; and (iii) in-patient death, defined as mortality occurring during the hospital stay.

For model development, clearly identified unplanned surgeries were included in the training outcome to reflect true deterioration and to modestly mitigate class imbalance by increasing the number of positive events. However, we did not use unplanned surgery when testing performance because the dataset did not reliably distinguish planned from deterioration-driven procedures. Only cases that could be confidently verified as unplanned and deterioration-related were incorporated during training; all others were excluded from the test outcome.

The study size was determined by the availability of routinely collected electronic health record data during the study period. We included all eligible adult inpatients to maximise the number of deterioration events, to capture a wide case-mix of patients, and to ensure stable model estimation. The start of the study period was selected to minimise the confounding effects of post-COVID operational recovery, while the end date corresponded to the latest date for which complete, validated data were available.

The data were split into training and validation cohorts using a temporal design to enable conservative model evaluation (Table 1). The training set included patients admitted between 1 January 2022 and 31 July 2024 (n = 32,787; deterioration rate = 6.2%), while the temporal validation set included patients admitted between 1 August 2024 and 31 October 2024 (n = 2577; deterioration rate = 1.9%). The validation cohort was defined as the most recent calendar period to mimic prospective model deployment, rather than through random sampling. As a result, the validation period, being more recent, had a lower deterioration rate than the training period, thereby providing a more conservative estimate of performance.

The prospective test cohort comprised patients admitted between 1 November 2024 and 31 January 2025 (n = 2625; deterioration rate = 3.3%). To assess the stability of the model performance over a longer timeframe, we conducted an informal temporal validation by extending the test period to 30 September 2025 (n = 11,048, deterioration rate = 2.8%) (Table A3).

### 2.4. Data Preprocessing

The data were extracted from the EPR as three separate tables: vital signs, laboratory tests, and clinical indicators. Following independent cleaning and addressing missing values, the tables were merged using the patient’s Visit ID as the unique identifier. Missing data were handled in the preprocessing pipeline without excluding patients, thereby reducing the risk of complete-case bias.

Each table contained multiple rows per patient, representing observations recorded at different time points during their hospital stay. To standardise the timing, observation times were rounded to the nearest hour. In cases where multiple observations were recorded within the same hour, the mean or maximum value was used, depending on the nature of the variable (for further details, see Appendix A.1). For each hour, missing values were initially filled forward using the most recent available observation to ensure temporal continuity. For any remaining missing values after forward-filling, a nearest neighbour imputation method was applied. This method leveraged data from similar patients, identified based on shared non-missing attributes, to estimate missing values.

Among vital signs, missing data rates were all below 0.05%, while supplemental oxygen percentage had 2.16% missing data. In laboratory tests, BNP (B-type Natriuretic Peptide) had the highest rate of missingness at 92%, whereas all other laboratory tests had missing rates below 15%. For laboratory tests with a high proportion of missing values, a normal reference value was imputed, based on the assumption that these tests were not performed because they were not clinically indicated. The higher rate of missing data in laboratory tests was primarily because not all tests are routinely performed for every patient.

Despite its high missingness, BNP was retained as a predictor because it reflects cardiac and haemodynamic stress frequently associated with deterioration and its absence often reflects lack of clinical indication rather than data quality loss [11]. However, to ensure robust model performance, we performed a sensitivity analysis by refitting the model without BNP. Model performance and hazard estimates for key covariates were materially unchanged, indicating that BNP is not required for robust model performance.

For clinical indicators such as palliative care flag and pain scores, missing values were assumed to indicate the absence of the condition or attribute. For instance, a missing palliative care flag was treated as zero, assuming the patient was not in palliative care. For frailty scores, which had 13% missing data, the median frailty score for patients within the same age group was used as the imputed value.

Appendix A.2 provides a detailed overview of the imputation techniques used for each variable with missingness, along with the percentage of missing data.

### 2.5. Feature Engineering

Predictors were selected based on their established relevance in previous early warning systems (EWS) and on clinical consensus regarding variables that reflect evolving physiological deterioration [8,9,10]. The final set of predictors comprised vital signs, laboratory tests, and clinical indicators that are routinely and reliably recorded in the EPR. Each variable was included both as its current value and, where clinically meaningful, as derived features representing the minimum, maximum, and trend over the preceding 12 h to capture temporal dynamics. The NEWS2 score and its 12-h trend were also included given their widespread clinical use and known association with deterioration risk. Table 2 lists all predictors used for model training.

Diagnosis and comorbidity are important predictors of patient outcomes, but these variables evolve during admission and were not consistently available in structured form. During hospital stay, comorbidities and diagnoses are often recorded only in ward round notes, which are unstructured free text and lack standardisation, making reliable extraction difficult. Final coded diagnoses and comorbidities are typically assigned retrospectively by clinical coders after discharge. For these reasons, and to reduce potential sources of bias due to inconsistencies in extraction, diagnosis and comorbidity were excluded from model training.

For completeness, we minimised bias by including all eligible patients during the study period, thereby reducing the risk of selection bias. Missing data were handled using a structured imputation pipeline to avoid complete-case bias. Diagnosis and comorbidity variables were excluded due to inconsistent availability in structured form, mitigating potential misclassification bias. Finally, a temporally separated validation dataset reduced the risk of optimistic performance estimates.

### 2.6. Model Selection

The training dataset exhibited significant class imbalance, with only 6.2% of patients experiencing a deterioration event. We evaluated several classification approaches, including logistic regression, random forest [12], and XGBoost [13], alongside common class-balancing techniques (oversampling, undersampling, and synthetic data generation with SMOTE [14]). These methods yielded inferior performance compared with NEWS2 and were therefore not pursued further. The best performance was achieved with a Cox proportional hazards model incorporating time-varying covariates. This approach naturally accounts for censoring, a frequent issue in clinical data when deterioration has not occurred by the end of observation and leverages the time-to-event structure of the data to provide more nuanced predictions than traditional classifiers.

### 2.7. Cox Proportional Hazard Model with Time-Varying Covariates

The Cox proportional hazards model is a widely used statistical approach in survival analysis, particularly suited for time-to-event data; however, to our knowledge, it has not been applied to clinical deterioration prediction. Unlike traditional classification models, which provide binary predictions, the Cox model estimates the hazard or risk of an event occurring at a given time, allowing for a more dynamic assessment of patient deterioration [15]. Time-varying covariates were incorporated to ensure that risk estimates reflected the evolving physiological state of patients rather than static baseline values [16]. This allowed the Cox model to update the hazard of deterioration dynamically as new observations were recorded. The implications of using time-varying covariates for distinguishing stable chronic abnormalities from true deterioration, and how this compares with the behaviour of NEWS2, are considered further in the Discussion.

A key assumption of the Cox model is that predictors maintain a linear relationship with the logarithm of the hazard function. However, many physiological variables follow nonlinear trajectories over time, which can violate this assumption. To better capture temporal dynamics while preserving linearity in the model, we performed feature engineering: each vital sign was expanded into three additional features (minimum, maximum, and trend over the last 12 h). For example, in addition to the current respiratory rate, we included the maximum and minimum values observed in the past 12 h, and a trend feature defined as the difference between the latest and 12-h-earlier measurements.

In addition, we explored functional transformations of the covariates (logarithmic, quadratic, and spline terms) to address potential nonlinearities and evaluated time-dependent coefficients using interactions with time terms. These did not improve model performance, so the engineered features were retained but no further transformations were applied.

Backward feature selection was used to identify the optimal set of predictors for the model. The selection process utilised Akaike Information Criterion (AIC) to balance model complexity and fit. Features were iteratively removed, and the model was re-evaluated at each step to ensure that the final set of predictors maximised predictive performance. The final Cox model was fitted using these transformed predictors (Table 2). Models were deployed using R version 4.2.2 with package Survival [17].

The final model was tested for violations of the proportional hazard assumption using Schoenfeld residuals. Several predictors demonstrated evidence of non-proportional hazards, which is expected in short-term, dynamic physiological data. Consistent with recent studies showing that moderate proportional-hazards violations generally have little impact on the predictive calibration of Cox-based models [18], and given that our primary aim is accurate 12-h risk prediction rather than estimation of time-constant effects, we prioritised discrimination and out-of-sample performance over strict proportional hazard adherence. Accordingly, estimated hazard ratios should be interpreted as average short-term associations with deterioration risk within the prediction window, rather than as constant effects operating over a patient’s entire hospital stay.

### 2.8. Validation and Alert Threshold

To utilise the model’s risk output in predicting patient deterioration and to create an intuitive clinical tool, we designed a tiered “traffic light” alert system, in which amber and red alerts represented increasing levels of risk. Predictions were generated from the most recent patient observations, and alerts were triggered when predicted risk exceeded the pre-defined amber or red thresholds. The model outputs a continuous, unitless prognostic score corresponding to the Cox model’s linear predictor, which increases monotonically with predicted short-term risk of deterioration and is recalculated dynamically as new patient observations become available.

The model was designed to predict deterioration 0–24 h before the event. Each prediction was assigned to either the 0–12 h or 12–24 h window. Accuracy metrics (recall, precision, and F1 score) were calculated across a range of model and NEWS2 thresholds (7–11). Predictions were classified as true positives (TP), false positives (FP), false negatives (FN), or true negatives (TN) according to the following rules: a TP was a correct prediction of deterioration within the 12–24 h window; a FP was a prediction of deterioration when none occurred; a FN was a missed deterioration within the 0–12 h window; and a TN was a correct prediction of no deterioration. Importantly, predictions below the threshold in the 12–24 h window were not considered false negatives, as the 12 h window was regarded as the hard limit for required predictions, while the 12–24 h window allowed for early warning without penalty. The 12-h threshold was selected to align with NICE guidance for inpatient monitoring, which recommends a minimum observation frequency of once every 12 h for patients with a NEWS2 score of 0 [19]. Operationally, this guidance implies that updated physiological observations should be available at least within each 12 h cycle for stable inpatients. As such, failure to raise an alert within 12 h of deterioration was considered a missed prediction, whereas alerts generated 12–24 h in advance were treated as early warnings without penalty.

For comparability, the same classification rules were applied to both the model and NEWS2. Model and NEWS2 performance were assessed against ICU transfers and in-patient deaths. To facilitate comparison with NEWS2, model thresholds were mapped to approximate NEWS2 scores by aligning precision and recall across the validation dataset, allowing equivalence between model outputs and NEWS2 thresholds (Table A2). The mapping demonstrated that a model risk score of 20 yielded performance comparable to NEWS2 ≥ 7. For operational deployment we selected more conservative alert thresholds to prioritise precision and reduce alert burden. The amber threshold was therefore set at a model risk score of 50, as a step above the NEWS2 equivalent performance, while the red threshold was set at a risk score of 80 to maximise precision.

Importantly, the comparison between the model and NEWS2 performance is based solely on ICU transfers and in-patient deaths. Emergency surgeries were excluded from the analysis as they range from minor to major procedures and were not labelled within the dataset. Including surgeries would require careful filtering on a case-by-case basis, making them unsuitable for this analysis.

### 2.9. Proximity of Alerts to Event

A primary aim in the model development was a reduction in clinician alert fatigue through producing a higher precision model. To assess this, we measured the proportion of alerts by lead-time to deterioration. For each alert, we located the next deterioration event for that patient and computed the time difference in hours between the alert timestamp and the event timestamp. If multiple events occurred, we used the first one after the alert. In this respect we measured the proportion of alerts as a function of hours before deterioration event. Alerts not followed by any deterioration during the admission were treated as no-event alerts and excluded from this figure—these are counted as false positives.

## 3. Results

### 3.1. Validation Model Performance

**Figure 1:**
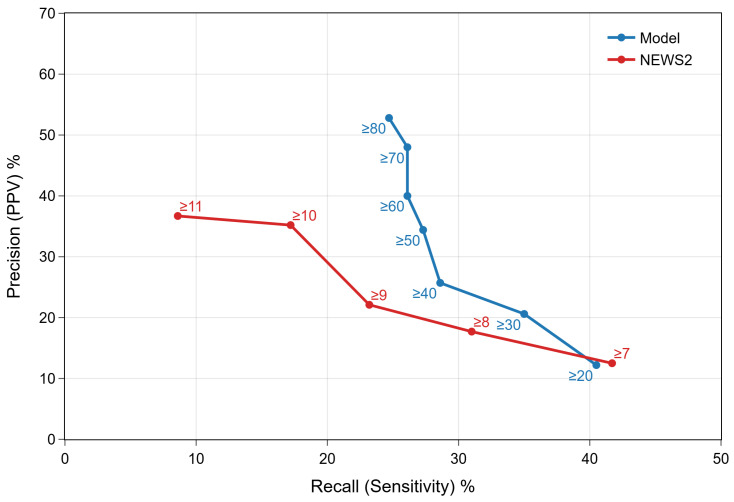
Precision-recall curves for the validation dataset (1 August 2024–31 October 2024), comparing the proposed model with NEWS2 across decision thresholds. The model’s amber alert (≥50) achieved 27% recall and 34% precision, the red alert (≥80) achieved 25% recall and 53% precision, whereas NEWS2 ≥7 achieved 42% recall with 13% precision.

The performance of our selected model was evaluated on a validation dataset of 2577 patients (1.9% with a deterioration event; Table 1) presenting to the Royal Albert Edward Infirmary, WWL (1 August 2024–31 October 2024). Using the precision–recall curve (Figure 1), we chose operating points for a tiered traffic-light system. Model thresholds were mapped to approximate NEWS2 scores by aligning operating characteristics across the validation dataset (Table A2). The red threshold was set at 80 to maximise precision and minimise unnecessary alerts. The amber threshold was set at 50 as a pragmatic step above the NEWS2 emergency response threshold (NEWS2 = 7) to facilitate use alongside NEWS2 rather than replace it. Empirically, a model score of ~50 corresponds to a NEWS2 of ~8.5, and 80 to ~9; a threshold near ~20 would have aligned with NEWS2 of 7 but was rejected due to materially lower precision and higher alert burden. At the red alert threshold, the model achieved a precision approximately four times greater than NEWS2 for identifying patients requiring emergency response (53% and 13%, respectively).

### 3.2. Log Hazard Ratios of Included Features

**Figure 2:**
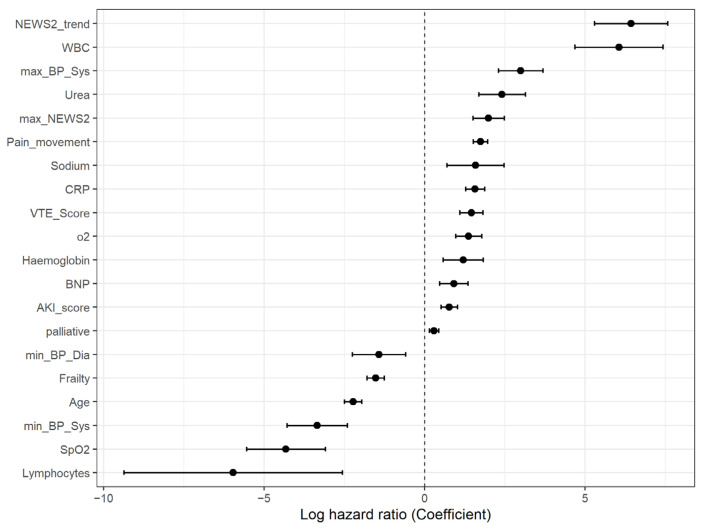
Forest plot of log hazard ratios (coefficients) means (dots) and 95% confidence intervals (lines) of the final Cox model. Variables are ordered by coefficient; positive coefficients (right of zero) indicate higher instantaneous risk per unit increase; negative coefficients indicate lower risk. Estimates reflect adjusted associations within the model (influenced by variable scaling and correlation) and, given evidence of non-proportional hazards, should be interpreted as time-averaged effects rather than strictly time-constant hazard ratios.

Figure 2 shows log hazard ratios from the final Cox model. Clinically intuitive signals stand out: lower SpO_2_, higher Urea, and higher NEWS2 together with a strongly positive NEWS2_trend that captures accelerating physiological instability deviate sharply from 0 and align with short-term (12 h) risk. The apparently counter-intuitive negative coefficients for age and frailty likely reflect cohort context, the population is predominantly older and frail, and short-horizon events are driven more by current physiology than baseline vulnerability. Moreover, ceilings of care (reduced probability of ICU transfer among the most frail/older patients) can lower the observed rate of deterioration event in the next 12 h even after adjusting for acute variables and palliative status.

Importantly, Cox model coefficients reflect conditional associations within the multivariable model and should not be interpreted as measures of feature importance or standalone predictive contribution. Coefficient magnitudes depend on variable scaling and correlations, and given the presence of non-proportional hazards, represent time-averaged effects rather than constant hazard relationships.

### 3.3. Temporal Risk Around Deterioration Events

**Figure 3:**
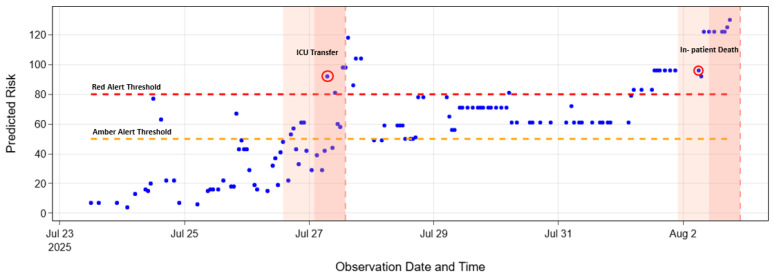
Example patient trajectory of model-predicted risk compared with NEWS2 across two deterioration events. Model-predicted risk (blue) increased steadily in the 24 h prior to the first deterioration event (ICU transfer), crossing the amber (≥50) and red (≥80) alert thresholds in close temporal proximity to the deterioration event (red circle). Following ICU admission, the predicted risk declined toward baseline, reflecting transient stabilisation, before rising again several days later to a sustained red-alert level that preceded a second deterioration event (in-patient death) within 36 h.

Figure 3 illustrates the temporal pattern of the model-predicted risk score for a representative patient who experienced deterioration events. The Cox model’s estimated hazard rose gradually in the 24 h preceding deterioration, with the risk score crossing the amber alert threshold (≥50) more frequently as the event approached. The first red alert (≥80) was triggered within 12 h of ICU transfer. Following ICU admission, the patient’s predicted risk declined to amber levels, reflecting transient stabilisation, before rising again several days later to a sustained red-alert level that preceded in-patient death within 36 h. In contrast, NEWS2 triggered only during the lead-up to the first deterioration event and did not trigger for the second. Between the first and second deterioration events, all NEWS2 measurements remained below the emergency alert threshold (NEWS2 < 7), including at the time of the second event (Supplementary Table S1 provides all NEWS2 and model predictions).

### 3.4. Live Model Performance

**Figure 4:**
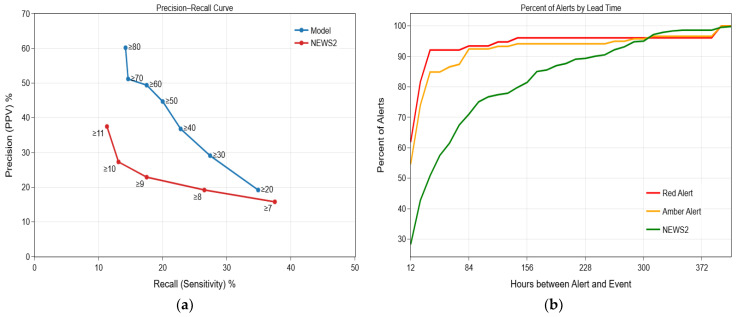
Live dataset (1 November 2024–31 January 2025). (**a**) Precision-recall curves for the live dataset, comparing the proposed model with NEWS2 across decision thresholds. The model’s amber alert (≥50) achieved 20% recall and 45% precision, the red alert (≥80) achieved 14% recall and 60% precision, whereas NEWS2 ≥7 achieved 38% recall with 16% precision. (**b**) Cumulative distribution of alerts by alert type (red, amber, and NEWS2) relative to the time before clinical deterioration; 82% of red alerts were triggered within 24 h of the event, compared to 74% of amber alerts and 43% of NEWS2 alerts.

The model was deployed in a live hospital setting and tested prospectively on 2625 patients admitted between 1 November 2024 and 31 January 2025 (Table 1). During this period, 86 deterioration events (3.3%) were recorded. The amber alert achieved a recall of 20% and a precision of 45%, while the red alert achieved a recall of 14% and a precision of 60% (Figure 4a). In comparison, the NEWS2 score at a threshold of ≥7, ≥8, and ≥9 achieved greater recall (38%, 27%, and 18%, respectively), but significantly lower precision (16%, 19%, and 23%, respectively).

A key decision in the development of this model was a reduction in the frequency of unnecessary alerting. Figure 4b demonstrates that a majority of red and amber alerts are within 24 h of a deterioration event (82% and 74%, respectively) whereas only 43% of NEWS2 alerts are within this period. Further support for the precision of our model and reduction in unnecessary alerts is demonstrated by the relatively minimal red and amber alerts generated before 84 h pre-deterioration (7% and 8%, respectively), compared with NEWS2 (29%).

### 3.5. Informal Evaluation Extension

**Figure A1:**
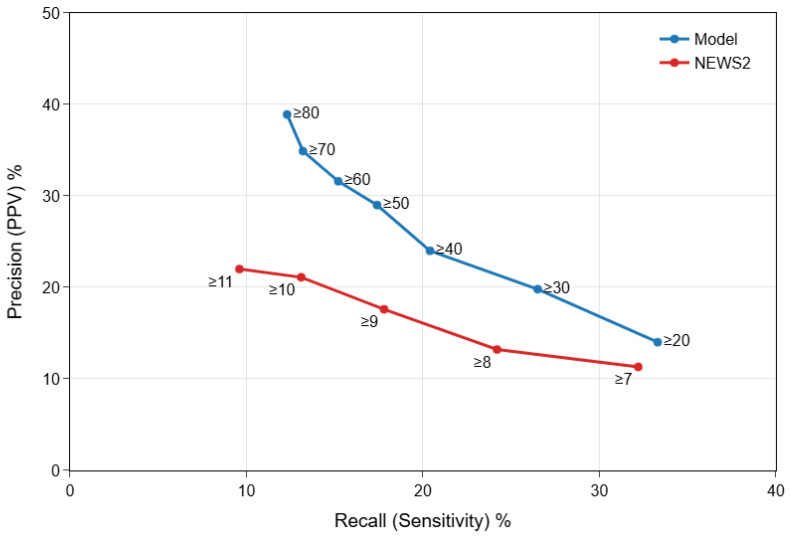
Precision-recall curves for the extended dataset (1 November 2024–30 September 2025), comparing the proposed model with NEWS2 across decision thresholds. The model’s amber alert (≥50) achieved 17% recall and 29% precision, the red alert (≥80) achieved 12% recall and 39% precision, whereas NEWS2 ≥7 achieved 32% recall with 11% precision.

To assess model stability beyond the original test period (1 November 2024 to 31 January 2025), the evaluation was informally extended to include data up to 30 September 2025 (n = 11,048, 2.8% deterioration events; Table A3). Model performance during the extended period remained consistent with the improvements observed in the pre-specified test dataset, showing substantially higher precision than NEWS2 across all emergency response thresholds (Table A4, Figure A1).

At the amber alert threshold, the model achieved a recall of 17% and precision of 29%, while at the red threshold recall was 12% and precision 39%. Over the same period, NEWS2 achieved recall and precision of 32% and 11%, respectively. Notably, a model threshold of ≥20 outperformed the standard NEWS2 threshold of ≥7, suggesting improved discrimination across all clinically relevant NEWS2 levels.

**Figure A2:**
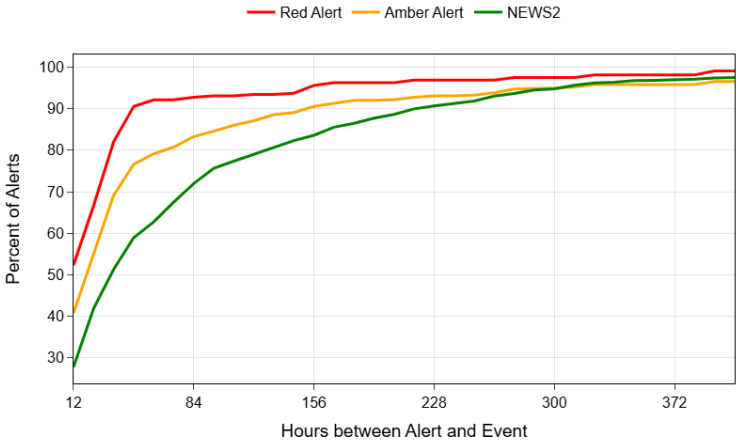
Percentage of alerts triggered as a function of hours between the alert and the deterioration event for the extended dataset (1 November 2024–30 September 2025). The plot shows the cumulative distribution of alerts by alert type (red, amber, and NEWS2) relative to the time before clinical deterioration; 67% of red alerts were triggered within 24 h of the event, compared to 55% of amber alerts and 42% of NEWS2 alerts.

Temporal alignment of alerts also remained stable: the majority of red (67%) and amber (55%) alerts occurred within 24 h of deterioration, compared with 42% for NEWS2. Conversely, only 7% of red and 17% of amber alerts were triggered more than 84 h before the event, compared with 28% for NEWS2 (Figure A2). These findings support the model’s ongoing precision and temporal proximity to deterioration events during extended deployment.

## 4. Discussion

This study set out to develop an early warning system (EWS) capable of predicting patient deterioration with greater precision than the conventional NEWS2 score. Using a Cox proportional hazards model with time-varying covariates, we sought to capture the dynamic nature of patient physiology and provide calibrated alerts. In our live hospital deployment, NEWS2 demonstrated higher recall, identifying a larger proportion of deteriorating patients, but this came at the expense of markedly lower precision and a substantially higher alert burden (Figure 4). In contrast, our model’s red alert threshold achieved a threefold improvement in precision over NEWS2 (16% vs. 60%), while amber alerts provided intermediate recall. This trade-off is clinically important: frequent false alarms contribute to increased workload, unnecessary disruption to patients, erosion of trust in EWS, and alert fatigue, a well-documented concern in clinical monitoring [7]. By reducing unnecessary alerts while maintaining temporal proximity to true events, our model may support more timely and actionable clinical responses. Although the planned evaluation period concluded in January 2025, an informal extension of the analysis through September 2025 demonstrated stable model performance and maintained superiority in precision over NEWS2 (Figure A1 and Figure A2), suggesting robustness to evolving case-mix and operational conditions.

A key advantage of the Cox framework with time-varying covariates is its ability to model deterioration relative to an evolving patient baseline. Unlike NEWS2, which repeatedly triggers alerts for persistently abnormal but stable parameters (e.g., patients admitted with chronically high respiratory rate), our model updates hazard estimates only when meaningful changes occur. Indeed, Figure 3 displays the power of our model—the model-derived risk score returned to a new baseline below red-alert tier after clinical stabilisation, suggesting our model more accurately tracks true deterioration than the static score of NEWS2. In our setting, 82% of red alerts and 74% of amber alerts occurred within 24 h of deterioration, compared to only 43% of NEWS2 alerts, many of which fired more than 84 h before the event (Figure 4b and Figure A2).

Direct comparisons with other published early warning models, such as eCART and the Advanced Alert Monitor (AAM), are constrained by differences in study design, patient populations, and endpoints. Nevertheless, these studies provide useful context. For example, eCARTv5 achieved an AUROC of 0.895, outperforming AAM (AUROC 0.82) in predicting deterioration [9,10]. However, AUROC is limited in imbalanced datasets like ours (3.3% event rate) because it is heavily influenced by the abundance of true negatives. Precision–recall (PR) curves provide a more informative assessment in this context [20,21]. At comparable levels of precision, our model achieved higher recall than both eCARTv5 and AAM in the high-precision operating range (e.g., 42% precision with 18% recall at the amber threshold). These results suggest our model performs particularly well in scenarios where reducing false alarms is a priority, even if this comes at the cost of lower recall at higher thresholds.

From a clinical perspective, the high precision of the red alert threshold suggests that our model may be particularly useful in confirming deterioration, helping clinicians focus on patients with the highest probability of imminent deterioration. However, the lower recall at this threshold indicates that some deteriorations will be missed if the red alert is used in isolation. Integrating both amber and red tiers offers a stratified approach: amber alerts can prompt increased monitoring, while red alerts provide high-confidence triggers for emergency intervention. Importantly, this positions our tool not as a replacement for NEWS2 but as a synergistic layer—NEWS2 provides broad recall, while our model’s tiered alerts sharpen precision, supporting escalation decisions and reducing alert fatigue.

Local feedback from the Critical Care Outreach Team and acute medical consultants at WWL supports this interpretation, highlighting the potential of combining NEWS2 and model-based alerts in daily practice. Clinicians reported that the model’s alerting behaviour aligned with their clinical perception of deterioration and that the interpretation was intuitive. In particular, they appreciated the model’s behaviour following deterioration and clinical stabilisation, in which predicted risk returned to a new baseline, which was perceived as distinct from NEWS2’s tendency to repeatedly flag persistently abnormal but stable physiology. This behaviour may support more meaningful clinical responses by distinguishing new deterioration from previously recognised risk, thereby reducing redundant alerts and reinforcing clinician confidence in escalation decisions.

Notably, when the analysis was informally extended to 30 September 2025, the model demonstrated consistent performance and outperformed NEWS2 across all clinically relevant emergency response thresholds (Figure A1). Specifically, a model threshold of ≥20 surpassed the NEWS2 threshold of ≥7, and this advantage persisted at every NEWS2 score > 7. These findings suggest that, as the model was exposed to a broader and more recent cohort, its discrimination improved relative to NEWS2, strengthening confidence in its clinical utility. While NEWS2 may still serve as a universal early screening tool, our model’s stability and superior performance across all operating points indicate that it could ultimately supersede or refine the NEWS2 role in escalation pathways rather than merely complement it.

An additional strength of our approach lies in its potential to identify pre-symptomatic features of deterioration. By incorporating temporal features (e.g., trends, minima, maxima) and dynamically updating covariates, the model may detect subtle shifts in physiology before overt clinical thresholds are breached. This raises the possibility of intervention earlier in the deterioration trajectory, potentially improving patient outcomes. With the addition of richer variables such as continuous monitoring data, treatment records, or natural language processing of clinician notes, this capacity for pre-symptomatic detection could be further enhanced.

While promising, our study has limitations. Our deterioration definition was limited to ICU transfer and in-patient death; potential competing events such as cardiac arrest or defibrillation outside ICU/HDU, initiation of vasopressors/inotropes, etc., were excluded and should be explored in future work. The dataset was imbalanced, with only a small proportion of patients experiencing deterioration, although this reflects the clinical reality. Additionally, while the Cox model is interpretable and transparent compared to many machine learning approaches, assumptions such as proportional hazards require careful consideration and further testing.

The study was conducted in a single NHS Trust, and external validation is needed to assess generalisability across other hospitals and patient populations. Further, diagnoses and comorbidities were excluded as a pragmatic decision due to challenges in their timely and reliable extraction from data sources, which may influence model transportability across institutions. Model performance may be affected when applied to patient populations with markedly different diagnostic or comorbidity profiles from those of our training cohort. Conversely, reliance on routinely recorded physiological variables may enhance generalisability between hospitals by reducing dependence on local coding practices and retrospectively assigned diagnoses.

Multicentre evaluation across NHS Trusts with differing patient demographics, clinical workflows, and escalation practices is a necessary next step to formally assess transportability in diverse real-world settings. In parallel, successful clinical impact will depend on effective operationalisation within routine workflows. Novel early warning systems may face barriers to adoption, including alert fatigue, workflow disruption, and clinician trust. Early operational experience suggests positive clinician engagement with our novel EWS. Future operationalisation-focused work should extend this start by evaluating usability, acceptability, and behavioural impact when integrating the model alongside NEWS2 in daily practice.

## Figures and Tables

**Table 1:** Baseline characteristics of the training, validation, and test cohorts. Percentages are calculated within each cohort. Time periods for each cohort (training, validation, test, respectively) are as follows: 1 January 2022 to 31 July 2024; 1 August 2024 to 31 October 2024; 1 November 2024 to 31 January 2025.

Characteristic	Training (n = 32,787)	Validation (n = 2577)	Test (n = 2625)
Deterioration events, n (%)	2050 (6.2%)	49 (1.9%)	86 (3.3%)
– ICU transfer ^†^	1545 (75.4%)	10 (20.4%)	34 (39.5%)
– In-patient death	505 (24.6%)	39 (79.6%)	52 (60.5%)
Age, mean (SD)	69 (17)	69 (18)	73 (16)
– 18–25, n (%)	606 (1.8%)	46 (1.8%)	32 (1.2%)
– 26–40, n (%)	2373 (7.2%)	188 (7.3%)	177 (6.7%)
– 41–60, n (%)	5921 (18.1%)	436 (16.9%)	420 (16.0%)
– 61–80, n (%)	13,593 (41.5%)	1146 (44.5%)	1139 (43.4%)
– >80, n (%)	10,294 (31.4%)	761 (29.5%)	857 (32.6%)
Sex, n (%)			
– Male	15,543 (47.4%)	1225 (47.5%)	1195 (45.5%)
– Female	17,244 (52.6%)	1352 (52.5%)	1430 (54.5%)

^†^ ICU transfer: In the validation and test sets, counts reflect actual ICU/HDU transfers only. In the training set, counts also include unplanned surgeries that were incorporated into the composite deterioration outcome to modestly increase positive events. We count these together because many patients who have unplanned surgery are subsequently transferred to ICU/HDU post-operatively, so in validation/test they are captured under ICU/HDU transfers even when surgery is not separately labelled.

**Table 2:** Features used for model training, including vital signs, laboratory tests, and clinical indicators. CRP: C-reactive protein; WBC: white blood cell count; BNP: B-type natriuretic peptide; SpO_2_: peripheral oxygen saturation; Sup. O_2_%: supplemental oxygen percentage; NEWS2: National Early Warning Score 2; AKI score: Acute Kidney Injury severity (0 = no injury, 1 = mild; 2 = moderate; 3 = severe); VTE score: Venous thromboembolism risk (0–10; higher scores indicate greater risk); Frailty: Clinical Frailty Scale (1–9; higher scores indicate greater frailty); Pain score: Patient-reported pain during movement, rated 0–3 (0 = none, 3 = severe); Palliative care flag: Indicator of active palliative care management.

Feature	Type	Range	Q1 (25th Percentile)	Median	Q3 (75th Percentile)	Median For Negative Class †	Median For Positive Class †	Mean For Negative Class †	Mean For Positive Class †
CRP	Continuous	0.4–672	10.0	27.0	75.0	27.0	55.0	57.5	102.7
Hemoglobin	Continuous	1.0–253.0	105.0	120.0	133.0	120	126	119	123.7
Lymphocytes	Continuous	0–189.5	0.8	1.2	1.7	1.2	1.1	1.4	1.3
Sodium	Continuous	100–185	135.0	138.0	140.0	138.0	138.0	137.6	137.1
Urea	Continuous	1.8–107.0	4.5	6.3	9.1	6.3	6.7	7.7	9.6
WBC	Continuous	0.1–307.1	6.3	8.3	11.0	8.3	11.5	9.3	12.4
BNP	Continuous	35–35,000	350	350	350	350	350	806.5	903.9
AKI score	Discrete	0–3	0	0	0	0	0	0.1	0.3
VTE score	Discrete	0–10	1	2	2	2	2	1.8	1.7
Age	Discrete	19–104	64	76	84	76	64	72.6	61.6
Palliative care Flag	Binary	0 or 1	-	-	-	-	-	-	-
Frailty	Discrete	1–9	3	4	5	4	3	4.2	3.2
Pain score	Discrete	0–3	0	0	0	0	0	0.1	0.3
Sup. O_2_%	Continuous	0–100	0	0	0	0	0	1.0	4.7
SpO_2_	Discrete	20–100	96	96	98	96	96	96.3	96
Min BP Diastolic *	Discrete	25–190	60	65	75	65	65	66.6	65.7
Min BP Systolic *	Discrete	30–255	110	120	135	120	115	121.7	116.3
Max BP Systolic *	Discrete	35–280	125	140	155	140	140	142.4	142.6
Max NEWS2 *	Discrete	0–19	0	1	3	1	2	1.8	3.5
NEWS2 trend *	Continuous	−16–17	−0.5	0	0	0	0	−0.1	0.5

* represents minimum, maximum or trend values over the preceding 12 h. † positive class refers to all observations within the 12 h preceding a deterioration event, negative class refers to all the other observations.

## Data Availability

The datasets generated and/or analysed during the current study are not publicly available due to the data sharing rules at WWL, but synthetic forms are available from the corresponding author on reasonable request.

## Supplementary Materials

The following supporting information can be downloaded at https://www.mdpi.com/article/10.3390/jcm15051690/s1, Table S1: Observation Data for a Representative Patient.

## Abbreviations

The following abbreviations are used in this manuscript:

WWL	Wrightington, Wigan, & Leigh
NEWS2	National Early Warning Score 2
EPR	Electronic Patient Record
EWS	Early Warning System
ICU	Intensive Care Unit
HDU	High Dependency Unit
A&E	Accident & Emergency

## Appendix A

### Appendix A.1. Averaging Technique for Observations Within the Same Hour

To standardise the timing, observation times were rounded to the nearest hour. In cases where multiple observations were recorded within the same hour, the maximum or mean value was used, depending on the nature of the variable.

- Maximum Value: used for categorical or risk-related features where the highest recorded value within the hour is clinically significant. Features: NEWS2, Frailty, Pain score, VTE Score and AKI score.
- Mean Value: used for continuous variables to provide a representative measure of patient status within the hour. Features: O2, SpO2, Minimum BP (Diastolic), Minimum BP (Systolic), Maximum BP (Systolic), Max. NEWS2, NEWS2 Trend, CRP, Haemoglobin, Lymphocytes, Sodium, Urea, WBC and BNP.

### Appendix A.2. Missing Percentage and Imputation Technique for Each Feature

**Table A1:** Abbreviations and definitions: CRP: C-reactive protein; WBC: white blood cell count; BNP: B-type natriuretic peptide; SpO_2_: peripheral oxygen saturation; Sup. O_2_%: supplemental oxygen percentage; NEWS2: National Early Warning Score 2; AKI score: Acute Kidney Injury severity (0 = no injury, 1 = mild; 2 = moderate; 3 = severe); VTE score: Venous thromboembolism risk (0–10; higher scores indicate greater risk); Frailty: Clinical Frailty Scale (1–9; higher scores indicate greater frailty); Pain score: Patient-reported pain during movement, rated 0–3 (0 = none, 3 = severe); Palliative care flag: Indicator of active palliative care management.

Feature	Percentage Missing	Imputation Technique
Age	0	
Sup. O_2_%	2.16	Iterative Imputer (using KNN with 3 nearest neighbours)
SpO_2_	0.02	
BP Dia	0.03	
BP Sys	0.12	
AKI Score	10.2	
VTE Score	12.4	
Haemoglobin	5.8	
Lymphocytes	5.9	
Sodium	6.8	
Urea	6.8	
WBC	5.8	
NEWS2	4.09	Calculated using other vitals
CRP	13.7	Filled with reference value (10)
BNP	91.9	Filled with reference value (350)
Frailty	13.8	Median for age group
Pain movement	6.4	Filled with zero

## Appendix B

### Performance of the Validation Cohort

**Table A2:** Performance metrics of the validation cohort; 1 August 2024–31 October 2024 (n = 2577). Recall and precision were calculated at each emergency response threshold and risk scores spanning ≥20–≥80, for NEWS2 and our model, respectively; 49 (1.9%) deterioration events occurred during this period.

NEWS2			Model		
Threshold	Recall	Precision	Threshold	Recall	Precision
≥7	42%	13%	≥20	41%	12%
≥8	31%	18%	≥30	35%	21%
≥9	23%	22%	≥40	29%	26%
≥10	17%	35%	≥50	27%	34%
≥11	9%	37%	≥60	26%	40%
			≥70	26%	48%
			≥80	25%	53%

## Appendix C

### Appendix C.1. Baseline Characteristics of the Extended Evaluation Cohort

**Table A3:** Baseline characteristics of the extended evaluation cohort; 1 November 2024–30 September 2025 (n = 11,048). The table summarises cohort sizes, deterioration events and subtypes, age distribution, and sex. Percentages are calculated within each cohort.

Characteristic	n = 11,048
Deterioration events, n (%)	315 (2.8%)
– ICU transfer	146 (46.3%)
– In-patient death	169 (53.7%)
Age, mean (SD)	73 (16)
– 18–25, n (%)	158 (1.4%)
– 26–40, n (%)	841 (7.6%)
– 41–60, n (%)	1862 (16.9%)
– 61–80, n (%)	4641 (42.0%)
– >80, n (%)	3546 (32.1%)
Sex, n (%)	
– Male	5143 (46.6%)
– Female	5905 (53.4%)
Characteristic	n = 11,048
Deterioration events, n (%)	315 (2.8%)
– ICU transfer	146 (46.3%)
– In-patient death	169 (53.7%)
Age, mean (SD)	73 (16)

### Appendix C.2. Performance of the Extended Evaluation Cohort

**Table A4:** Performance metrics of the extended evaluation cohort; 1 November 2024–30 September 2025 (n = 11,048). Recall and precision were calculated at each emergency response threshold and risk scores spanning ≥20–≥80, for NEWS2 and our model, respectively; 315 (2.8%) deterioration events occurred during this period.

NEWS2			Model		
Threshold	Recall	Precision	Threshold	Recall	Precision
≥7	32%	11%	≥20	33%	14%
≥8	24%	13%	≥30	27%	20%
≥9	18%	18%	≥40	20%	24%
≥10	13%	21%	≥50	17%	29%
≥11	10%	22%	≥60	15%	32%
			≥70	13%	35%
			≥80	12%	39%
