# Supplementary material for: A High-Precision Time-Varying Survival Model for Early Prediction of Patient Deterioration: A Retrospective Cohort Study
Source: J Clin Med. 2026 Feb 24;15(5):1690. doi: 10.3390/jcm15051690 (PMC12985587; doi:10.3390/jcm15051690)
Supplement: Supplementary file 1 [file jcm-15-01690-s001.zip › jcm-4142023-supplementary.pdf]

Supplementary Data

Supplementary S.1

Observation data for a representative patient.

The patient experienced two deterioration events. The first event was an ICU transfer at 14:00 on 27 July 2025; within the 12 hours preceding this event, the earliest model-generated red alert was triggered at 07:00 on 27 July (Predicted Risk 92), with NEWS2 exceeding the emergency threshold for the first time at 10:00 (NEWS2 = 8). The second event was in-patient death at 22:00 on 2 August 2025, with the earliest red alert triggered at 05:00 on 1 August (Predicted Risk 83), 41 hours before the event, and remaining above the red-alert threshold thereafter. Between the first and second deterioration events, NEWS2 scores remained below the emergency threshold (NEWS2 <7). Additionally, there were time points outside the 12-hour prediction window when both measures transiently exceeded their respective alert thresholds.

Table S1. Observation data for a representative patient, showing Observation Date & Time, NEWS2, and Model-Predicted Risk.

Observation Date & Time	NEWS2	Predicted Risk	Observation Date & Time	NEWS2	Predicted Risk	Observation Date & Time	NEWS2	Predicted Risk
23/07/2025 12:00	1	7	26/07/2025 22:00	4	61	29/07/2025 22:00	5	71
23/07/2025 15:00	1	7	26/07/2025 23:00	5	42	30/07/2025 00:00	5	71
23/07/2025 22:00	1	7	27/07/2025 01:00	4	29	30/07/2025 02:00	5	71
24/07/2025 02:00	1	4	27/07/2025 03:00	5	39	30/07/2025 04:00	5	71
24/07/2025 05:00	2	13	27/07/2025 05:00	4	29	30/07/2025 05:00	5	81
24/07/2025 09:00	2	16	27/07/2025 06:00	5	42	30/07/2025 06:00	5	61
24/07/2025 10:00	2	15	27/07/2025 07:00	4	92	30/07/2025 08:00	5	61
24/07/2025 11:00	2	20	27/07/2025 09:00	4	44	30/07/2025 13:00	5	61
24/07/2025 12:00	2	77	27/07/2025 10:00	8	81	30/07/2025 14:00	5	61
24/07/2025 15:00	2	63	27/07/2025 11:00	4	60	30/07/2025 17:00	5	61
24/07/2025 17:00	1	22	27/07/2025 12:00	4	58	30/07/2025 21:00	5	61
24/07/2025 20:00	1	22	27/07/2025 13:00	5	98	31/07/2025 03:00	5	61
24/07/2025 22:00	1	7	27/07/2025 14:00	5	98	31/07/2025 05:00	5	72
25/07/2025 05:00	1	6	27/07/2025 15:00	5	118	31/07/2025 06:00	5	61
25/07/2025 09:00	2	15	27/07/2025 17:00	5	86	31/07/2025 08:00	5	61
25/07/2025 10:00	2	16	27/07/2025 18:00	5	104	31/07/2025 09:00	5	61
25/07/2025 11:00	2	16	27/07/2025 20:00	5	104	31/07/2025 13:00	5	61
25/07/2025 13:00	2	16	28/07/2025 01:00	5	49	31/07/2025 16:00	5	61
25/07/2025 15:00	3	22	28/07/2025 04:00	5	49	31/07/2025 17:00	5	61
25/07/2025 18:00	3	18	28/07/2025 05:00	5	59	31/07/2025 19:00	5	61

25/07/2025 19:00	3	18
25/07/2025 20:00	7	67
25/07/2025 21:00	5	43
25/07/2025 22:00	5	49
25/07/2025 23:00	4	43
26/07/2025 00:00	4	43
26/07/2025 01:00	3	29
26/07/2025 03:00	4	19
26/07/2025 04:00	3	16
26/07/2025 08:00	5	15
26/07/2025 10:00	7	32
26/07/2025 11:00	5	37
26/07/2025 12:00	5	19
26/07/2025 13:00	6	41
26/07/2025 14:00	6	48
26/07/2025 16:00	6	22
26/07/2025 17:00	7	53
26/07/2025 18:00	6	57
26/07/2025 19:00	5	43
26/07/2025 20:00	3	33
26/07/2025 21:00	6	61
28/07/2025 10:00	5	59
28/07/2025 11:00	5	59
28/07/2025 12:00	5	59
28/07/2025 13:00	5	50
28/07/2025 15:00	5	50
28/07/2025 16:00	5	50
28/07/2025 17:00	5	51
28/07/2025 18:00	5	78
28/07/2025 20:00	5	78
29/07/2025 05:00	5	78
29/07/2025 06:00	5	65
29/07/2025 07:00	5	56
29/07/2025 08:00	5	56
29/07/2025 10:00	5	71
29/07/2025 11:00	5	71
29/07/2025 13:00	5	71
29/07/2025 15:00	5	71
29/07/2025 17:00	5	71
29/07/2025 18:00	5	71
29/07/2025 19:00	5	71
29/07/2025 21:00	5	71
31/07/2025 20:00	5	61
01/08/2025 03:00	5	61
01/08/2025 04:00	5	79
01/08/2025 05:00	5	83
01/08/2025 08:00	5	83
01/08/2025 12:00	5	83
01/08/2025 13:00	5	96
01/08/2025 14:00	5	96
01/08/2025 15:00	5	96
01/08/2025 17:00	5	96
01/08/2025 19:00	5	96
01/08/2025 21:00	5	96
02/08/2025 06:00	5	96
02/08/2025 07:00	5	92
02/08/2025 08:00	5	122
02/08/2025 10:00	5	122
02/08/2025 12:00	5	122
02/08/2025 15:00	5	122
02/08/2025 16:00	5	122
02/08/2025 17:00	5	125
02/08/2025 18:00	5	130
